# Integrating Multi-Environment Phenotypes and Genome-Wide Variation to Evaluate Diversity and Identify Representative Germplasm in Specialty Maize

**DOI:** 10.3390/genes17050568

**Published:** 2026-05-17

**Authors:** Hui Wang, Zhixiong Zhao, Wen Xu, Pingdong Sun, Siyu Zhao, Jingtao Qu, Yinxiong Hu, Jihui Wei, Hongjian Zheng

**Affiliations:** 1 Shanghai Key Laboratory of Agricultural Genetics and Breeding, Shanghai Engineering Research Center of Specialty Maize, Crop Breeding and Cultivation Research Institution/CIMMYT-China Specialty Maize Research Center, Shanghai Academy of Agricultural Sciences, Shanghai 201403, China; 2 College of Agriculture, Xinjiang Agricultural University, Urumqi 830052, China

**Keywords:** specialty maize, agronomic traits, phenotypic diversity, population structure, genome-wide SNPs, core germplasm

## Abstract

Objectives: To facilitate the innovation and efficient utilization of specialty maize germplasm, this study aimed to systematically evaluate a panel of 222 inbred lines. The objective was to comprehensively characterize phenotypic variation, genetic diversity, and genotype–phenotype associations to screen for representative germplasm resources. Methods: We integrated Best Linear Unbiased Prediction (BLUP) values derived from multi-environment field trials with high-density whole-genome single-nucleotide polymorphism (SNP) data. Population structure and genetic diversity were analyzed, Mantel tests were conducted to assess genotype–phenotype correspondence, and a genome-wide association study (GWAS) was performed to identify significant loci. Results: The population exhibited substantial phenotypic variation, particularly in plant height and tassel traits, with distinct morphological differentiations among specialty types. Genetic diversity analyses revealed varying diversity levels among subpopulations. While Mantel tests indicated a weak overall genotype–phenotype correspondence, specific traits showed significant associations with genetic distance. GWAS successfully identified significant loci associated with plant height and tassel traits. Furthermore, population structure analysis revealed distinct genetic stratification corresponding to specialty types, albeit with a certain degree of admixture. Conclusions: By integrating multi-dimensional phenotypic and genomic profiles, a panel of highly diverse and representative candidate germplasm was identified. These findings provide a crucial theoretical basis for specialty maize breeding and the optimized utilization of germplasm resources.

## 1. Introduction

Maize is a globally important food and economic crop, with its broad ecological adaptability contributing to substantial genetic diversity [1]. Specialty maize, including sweet, waxy, and sweet–waxy types, has gained increasing market demand and cultivation area due to its specific nutritional quality (such as high sugar content in sweet maize and high amylopectin in waxy maize) and high added value, making it a key focus of maize breeding programs [2,3].

Genetic diversity forms the foundation for germplasm innovation. Recent studies using molecular markers such as SNPs have revealed significant population structure differentiation in specialty maize [4]. Moreover, our previous study conducted GWAS and genomic prediction analyses of key plant architecture traits in a combined sweet and waxy maize population, providing valuable insights into their genetic architecture [5]. However, as an initial step, our previous work was restricted to a specific population and solely focused on plant architecture, leaving kernel morphology and tassel architecture unexplored. Since different germplasm populations possess distinct genetic backgrounds and may harbor unique allelic variations, evaluating a novel population is crucial for capturing broader genetic diversity and validating genotype–phenotype associations. Furthermore, the inclusion of the increasingly important sweet–waxy maize type and the practical selection of representative core germplasm remained to be addressed. Consequently, the overall genetic landscape of specialty maize remains insufficiently characterized. Phenotypic variation in these crops is frequently influenced by complex genotype-by-environment interactions (G × E). Therefore, advanced statistical models, such as BLUP, are essential for accurately extracting genetic signals from multi-environment trials. Moreover, morphological traits such as kernel size and plant architecture not only determine market appearance but also directly impact final yield and lodging resistance. The integration of phenotypic and genotypic data has been shown to enable more precise elucidation of germplasm structure [6]. Simultaneously, GWAS leveraging high-density markers have emerged as a powerful tool for dissecting the genetic architecture of key agronomic traits. Nevertheless, current phenotypic evaluations of specialty maize have predominantly emphasized yield and quality traits, with limited consideration of key agronomic traits directly affecting market appearance (kernel morphology), lodging resistance and mechanization adaptability (plant architecture), and tassel traits. Additionally, strategies for core germplasm selection driven by combined phenotype–genotype analyses remain underdeveloped.

To address these gaps, the present study employed 222 specialty maize inbred lines encompassing sweet, waxy, and sweet–waxy types, integrating multi-dimensional phenotypic data on kernel, plant, and tassel traits with genome-wide SNP information to systematically assess genetic diversity and population structure. The objectives of this study were to: (1) characterize phenotypic variation in key agronomic traits in specialty maize; (2) reveal patterns of genetic differentiation among different specialty maize types; and (3) construct core germplasm based on combined phenotype–genotype analysis. The findings are expected to provide theoretical guidance and high-quality resources for genetic improvement and efficient breeding of specialty maize.

## 2. Materials and Methods

### 2.1. Materials and Planting

The specialty maize inbred lines used in this study were developed through multiple generations of selfing and purification at our institute. A total of 222 inbred lines were selected as experimental materials, including 123 sweet corn (SHT1–SHT123), 82 waxy corn (SHN1–SHN82), and 17 sweet–waxy corn lines (SHTN1–SHTN17). Field trials were conducted across two contrasting environments. The first trial was carried out in 2024 at the Zhuanghang Comprehensive Experimental Station, Shanghai (30°53′ N, 121°39′ E), representing a typical spring maize ecological zone (sown in April and harvested in July 2024). The second trial was conducted as a winter nursery at Lingshui, Hainan (18°34′ N, 110°04′ E; sown in December 2024 and harvested in March 2025). Shanghai experiences a humid subtropical climate with four distinct seasons, whereas Lingshui features a tropical climate with warm temperatures year-round. These substantial climatic and seasonal differences validate their use as distinct environments for evaluating genotype-by-environment interactions in the present study.

### 2.2. Experimental Design and Management

The experiment was arranged in a randomized complete block design. Each plot consisted of two rows measuring 3.0 m in length, with a row spacing of 0.70 m and an intra-row spacing of 0.25 m. Each inbred line was replicated three times. All materials were managed under local standard agronomic practices to ensure the consistency and reliability of the growing conditions. At the silking and pollination stage, 10 plants exhibiting uniform growth vigor and synchronized flowering were selected from each plot for manual self-pollination using bags. Depending on climatic conditions and grain development status, ears were harvested uniformly at the physiological maturity stage.

### 2.3. Investigation of Agronomic Traits

The following ten key agronomic traits were investigated: kernel length (KL), kernel width (KW), and length-width ratio (LWR) as grain traits; plant height (PH), ear height (EH), and ear position ratio (EPR) as plant architecture traits; and primary tassel branch number (TPN), tassel branch length (TBL), tassel length of the main axis above the lowest lateral branch (LUL), and length of upper tassel (LUT) as tassel morphology traits. The investigation of agronomic traits was carried out in stages, and the measurement methods were primarily based on the Guidelines for the Conduct of Tests for DUS-Maize (GB/T 19557.24-2018). PH and EH were measured one month after pollination. Six representative plants were systematically selected from each plot for manual measurement, and their mean value was used as the observed value for that plot. Tassel traits, including TBN, TBL, LUL, and LUT, were investigated at the full flowering stage using the same six plants mentioned above. Grain traits were determined at the physiological maturity stage. After maturation, all ears were harvested separately by plot. Three standard ears with plump grains and free from diseases and pests were randomly selected for natural air-drying and manual threshing. KL, KW, and LWR were measured using a digital maize seed testing machine (Model: YTS-MKT; Wuhan Gufeng Optoelectronic Technology Co., Ltd., Wuhan, China).

### 2.4. Statistical Data Analysis

To accurately estimate genetic effects and explicitly quantify the magnitude of genotype-by-environment (G × E) interactions, phenotypic data were analyzed using a linear mixed model (LMM). Given that the field trials were conducted in different locations across different years (Zhuanghang in 2024 and Lingshui in 2025), the “Environment” factor was explicitly defined as the combined effect of location and year (E1 and E2). Preliminary analysis confirmed no significant differences among the three blocks within each environment; therefore, the phenotypic values of the three blocks were averaged to obtain a single representative value per genotype per environment prior to model fitting.

To predict random genetic effects and calculate BLUPs, all effects except the overall mean were treated as random effects. The simplified linear mixed model was specified as follows: yik=μ+Ei+Gk+(GE)ik+εik. Where yik represents the phenotypic value of the k-th genotype in the i-th environment; μ is the overall mean; Ei is the fixed effect of the i-th environment; Gk is the random effect of the k-th genotype; (GE)ik is the random interaction effect between the k-th genotype and i-th environment; εik is the residual error.

The BLUPs for each trait were then extracted to obtain phenotypic values adjusted for environmental noise. Based on these BLUP values, descriptive statistics including min, max, mean, SD, and CV were calculated for all 222 inbred lines across ten traits to assess phenotypic variation. These BLUP values were subsequently used for multivariate analyses. Additionally, one-way ANOVA was performed to test differences among groups, and Duncan’s multiple range test (*p* < 0.05) was applied for multiple comparisons. Preliminary data processing, descriptive statistics, and figure plotting were conducted using Origin 2024 software, while linear mixed model fitting, BLUP extraction, and multivariate statistical analyses were performed in R software (v4.3.0).

### 2.5. SNP Data Quality Control and Genetic Diversity Analysis

To obtain the SNP genotype data for each inbred line, fresh young leaves were collected at the tenth leaf stage and sent to Novogene Bioinformatics Institute (Beijing, China) for DNA extraction and whole-genome resequencing. DNA extraction was performed following the company’s standard protocols. Clean reads were mapped to the B73 reference genome (RefGen_v5; Zm-B73-REFERENCE-NAM-5.0) using BWA-MEM. After quality filtering of the raw SNP data for all samples, a high-quality SNP dataset comprising 11,021,213 loci was obtained. For this dataset, per-sample sequencing depth at each SNP locus was calculated using bcftools (v1.19), and the average sequencing depth across all samples and loci was determined to assess coverage. Subsequently, VCFtools (v0.1.16) and PLINK 2.0 were used to calculate polymorphism information content (PIC), nucleotide diversity (π), observed heterozygosity (Het), and minor allele frequency (MAF) for each SNP locus in order to evaluate the genetic diversity and informativeness of the SNP dataset and provide a basis for downstream analyses such as genome-wide association studies (GWAS) and population structure analysis [7,8,9]. In addition, these metrics were calculated for each predefined subgroup, and the average values were compared to assess differences in genetic diversity among groups.

### 2.6. Phenotypic Diversity and Trait Relationship Analysis

Based on the sample size (N) of each group, the optimal number of classes (k) for each trait was determined using Sturges’ formula. The calculated results were: k = 9 for the entire population, k = 8 for the sweet corn group, k = 7 for the waxy corn group, and k = 5 for the sweet–waxy corn group. Subsequently, the trait observations were divided into k continuous non-overlapping intervals using the equidistant classification method. The sample frequency (pi) for each interval was calculated, and the Shannon-Wiener index formula was used to calculate the diversity index [10]. In addition, to explore the genetic relationships among the 222 fresh maize inbred lines and the association patterns among the ten traits, hierarchical clustering was performed on both samples and traits based on standardized data, and a bi-clustering heatmap was constructed to visualize the simultaneous clustering of samples and traits. Pearson correlation coefficients were also calculated to analyze the correlations among the ten traits.

### 2.7. Assessment of Genetic and Phenotypic Variation via PCA and Clustering

To comprehensively assess the genetic and phenotypic diversity of the germplasm, we conducted independent principal component analyses (PCAs) using SNP genotypes and the best linear unbiased prediction (BLUP) values for 10 agronomic traits. On the phenotypic front, a loading biplot was generated to quantify how strongly each trait drove the variance in the phenotypic principal components. To further resolve the phenotypic differentiation patterns, a *K*-means clustering algorithm was executed on the *Z*-score standardized BLUP values. The optimal cluster number (*K*) was rigorously identified through a dual evaluation of the elbow method and silhouette scores. Ultimately, the unique agronomic profiles of the distinct germplasm groups were delineated by comparing the mean values of multiple traits across the resulting clusters.

### 2.8. Genotype–Phenotype Association Analysis

SNP genotype data were used to construct genotype distance matrices for all samples. A comprehensive phenotypic distance matrix and single-trait phenotypic distance matrices were calculated for 10 traits. The Mantel test was performed to assess correlations between the genotype distance matrix and both the overall and single-trait phenotypic distance matrices, in order to evaluate genotype–phenotype relationships [11]. For traits potentially associated with genetic distance, genome-wide association studies (GWAS) were conducted using the linear mixed model (LMM) implemented in GEMMA (v0.98.5). A kinship matrix was derived from the SNP data to account for relatedness among individuals. Model performance and statistical results were evaluated using QQ plots [12].

### 2.9. Population Structure Inference and NJ Tree Construction

The population structure of 222 inbred lines was analyzed using Structure software (v2.3.4) [13]. The number of clusters (K) was set from 1 to 10, and each K value was run 10 times independently to reduce stochastic variation. The optimal K was determined by comparing the likelihood distributions (lnP(D)) across different K values and the stability of individual assignment probabilities, and the Q matrix was visualized accordingly. Based on the SNP genotype data of the 222 inbred lines, a phylogenetic tree was constructed using the Neighbor-Joining (NJ) method [14]. A genetic distance matrix was first calculated between individuals, and the NJ tree was built by iteratively joining the nearest neighbor nodes.

### 2.10. Germplasm Evaluation and Core Collection Selection

Based on the pairwise genetic distance matrix of all inbred lines, the mean genetic distance (MNND) was calculated for each line to quantify its genetic uniqueness [15]. Ten traits were then selected (grain-related traits as positive indicators, and plant architecture and male inflorescence traits as negative indicators), and the CV of each trait was calculated as a weight. The positive and negative indicators were standardized accordingly. Subsequently, a comprehensive phenotypic score (D value) for each line was calculated using the TOPSIS method [16]. based on the standardized data. On this basis, MNND scores and TOPSIS phenotypic scores were integrated in a two-dimensional analysis, and a scatter plot of “MNND vs. TOPSIS scores” was generated to visually display the distribution of genetic uniqueness and phenotypic superiority. The top 50 lines based on TOPSIS D values were selected as phenotypically superior. Within this subset, a weighted composite score was calculated, with MNND contributing 20% and the TOPSIS score contributing 80%. Finally, the top 30 lines ranked by the composite score were selected to construct the core germplasm collection.

## 3. Results

### 3.1. Descriptive Statistics of Phenotypic Traits

Table 1 summarizes the main phenotypic traits and their descriptive statistics for the maize inbred line population. The results indicate that substantial variation exists in both kernel-related traits (e.g., KL and KW) and agronomic traits (e.g., PH, EH, and TPN). Notably, the ranges of PH and EH were 133.00 cm and 71.83 cm, respectively, indicating a wide variation in plant architecture within the population. In contrast, tassel-related traits (e.g., LUL and LUT) exhibited moderate-to-relatively high mean values. Detailed statistical parameters, including mean, SD, minimum, and maximum values for each trait, are presented in Table 1. All phenotypic data were based on BLUP values, and the proportion of phenotypic variance explained by different components is shown in Figure S1.

### 3.2. Phenotypic and Genetic Differentiation Among Subpopulations

**Figure 1:**
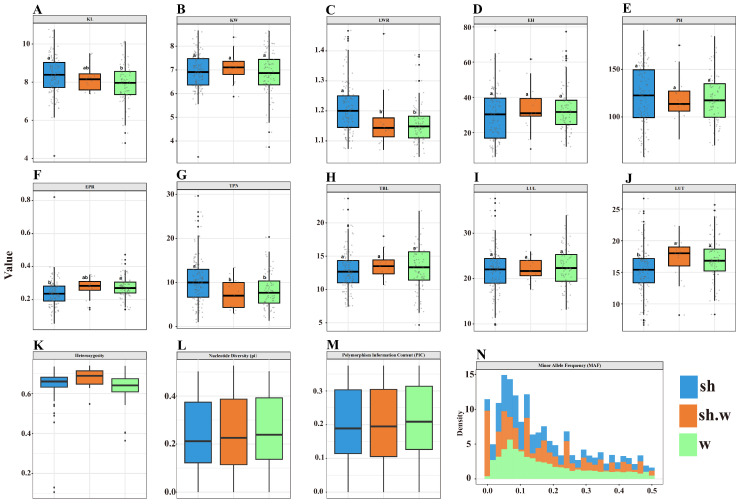
Comparison of phenotypic traits and genetic diversity parameters among three maize subpopulations. The three subpopulations include sweet corn (sh), sweet–waxy corn (sh.w), and waxy corn (w). Boxplots (**A**–**J**) show the distribution of ten phenotypic traits, including KL (**A**), KW (**B**), LWR (**C**), EH (**D**), PH (**E**), EPR (**F**), TPN (**G**), TBL (**H**), LUL (**I**), and LUT (**J**). Different letters above the boxplots indicate significant differences among subpopulations (*p* < 0.05). Panels (**K**–**M**) represent genetic diversity parameters, including heterozygosity (**K**), nucleotide diversity (π) (**L**), and polymorphism information content (PIC) (**M**). Panel (**N**) shows the distribution of minor allele frequency (MAF) in the three subpopulations.

To further characterize the variation within the population, the 222 specialty maize inbred lines were divided into three subgroups: sweet corn, waxy corn, and sweet–waxy corn. Significant differences in ten phenotypic traits and genetic diversity parameters were analyzed (Figure 1).

For phenotypic traits, distinct patterns of differentiation were observed among subgroups. For kernel-related traits, KL and LWR in sweet corn were significantly higher than those in waxy and sweet–waxy corn, whereas no significant difference in KW was observed among the three groups (Figure 1A–C). For plant architecture traits, no significant differences were detected in PH or EH among subgroups; however, EPR in waxy and sweet–waxy corn was significantly higher than that in sweet corn (Figure 1D–F). For tassel-related traits, TPN in sweet corn was significantly higher than that in waxy and sweet–waxy corn, while no significant differences were observed in TBL or LUL among subgroups. In addition, LUT in sweet corn was significantly lower than that in the other two subgroups (Figure 1G–J).

For genetic diversity parameters, distinct distribution patterns were observed among subgroups. The average heterozygosity followed the order: sweet–waxy corn > sweet corn > waxy corn (Figure 1K). In contrast, nucleotide diversity and polymorphism information content were highest in waxy corn, followed by sweet–waxy corn, and lowest in sweet corn (Figure 1L,M). Furthermore, the distribution of MAF showed that sweet–waxy corn consistently exhibited intermediate values between sweet and waxy corn (Figure 1N).

### 3.3. Phenotypic Diversity Analysis

**Figure 2:**
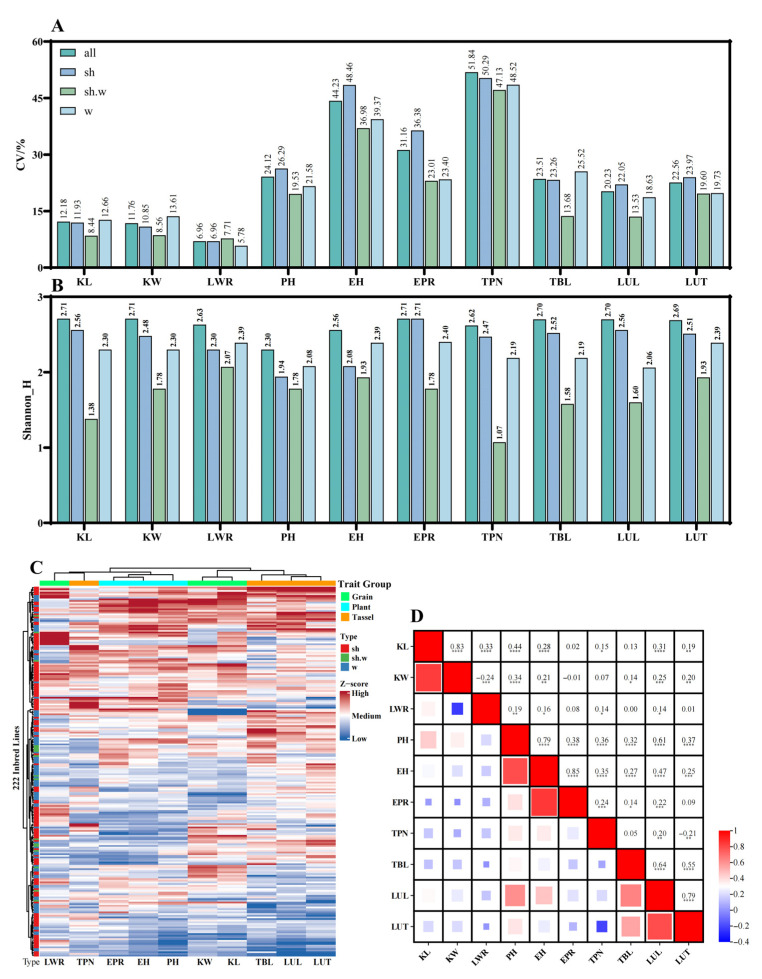
Phenotypic diversity and trait relationships in the maize inbred line population. (**A**) Coefficients of variation (CVs) of ten phenotypic traits in the whole population (all) and three subpopulations: sweet corn (sh), sweet–waxy corn (sh.w), and waxy corn (w). (**B**) Phenotypic diversity index based on the Shannon index for each trait across the population and subpopulations. (**C**) Hierarchical clustering heatmap of the 222 inbred lines based on standardized phenotypic data. Traits are grouped into Grain, Plant, and Tassel categories. (**D**) Pearson correlation matrix among the ten phenotypic traits. Color intensity represents the strength and direction of correlation, and the values indicate correlation coefficients. * *p* < 0.05; ** *p* < 0.01; *** *p* < 0.001; **** *p* < 0.0001.

The analysis of phenotypic variation across the 222 inbred lines revealed that EH and TPN exhibited the highest CV, reaching 44.23% and 51.84%, respectively. Among subpopulations, sweet corn showed higher CV values for EH, PH, TPN, LUL, and LUT compared to the other two groups, whereas the variation in KL and KW was slightly lower than that in waxy corn. In contrast, sweet–waxy corn exhibited the lowest CV values for most traits, except for LWR (Figure 2A).

The phenotypic diversity index based on the Shannon index indicated an overall high level of diversity in the population, ranging from 2.62 to 2.71. Among subpopulations, sweet–waxy corn consistently showed the lowest Shannon index across all ten traits, suggesting relatively limited phenotypic diversity. Sweet corn exhibited the highest diversity for most traits, except for LWR and EH, with the highest index observed for EPR (2.71) and the lowest for PH (1.94) (Figure 2B).

Hierarchical clustering based on all phenotypic traits grouped the ten traits into three major categories: Grain, Plant, and Tassel. The clustering results largely agreed with the predefined trait classifications, although LWR (Grain) and TPN (Tassel) were grouped together, indicating a potential association between these traits (Figure 2C).

Pearson correlation analysis further revealed the relationships among traits. Within the Tassel group, TPN showed significant negative correlations with TBL, LUL, and LUT, whereas TBL, LUL, and LUT were positively correlated with each other, with the strongest correlation observed between LUL and LUT (r = 0.79). Within the Plant group, all three traits were significantly positively correlated. In the Grain group, only KW and LWR showed a significant negative correlation (r = −0.24). Across trait groups, Grain traits were positively correlated with PH and EH but showed no significant correlation with EPR. Traits in the Plant and Tassel groups generally exhibited positive correlations, except for a weak association between EPR and LUT. At the individual trait level, KL and KW were positively correlated with LUL and LUT (Figure 2D).

### 3.4. Association Between Genotypic and Phenotypic Variation

**Figure 3:**
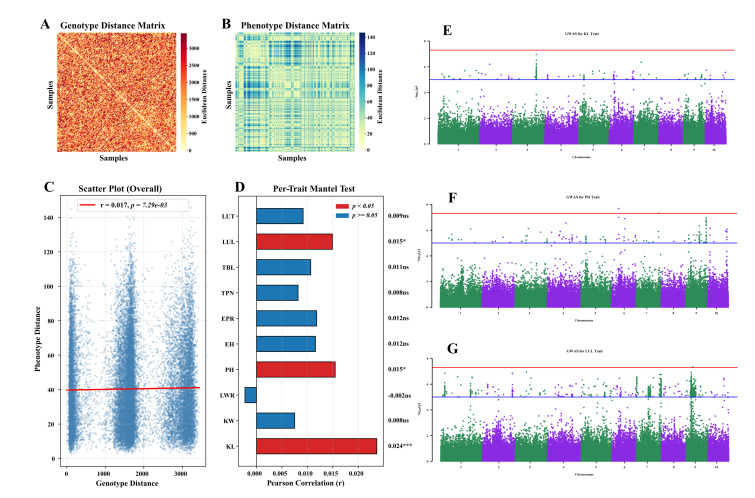
Genotype–phenotype association analysis. (**A**) Genotypic distance matrix. (**B**) Phenotypic distance matrix. (**C**) Mantel test between genotypic and phenotypic distances. (**D**) Per-trait Mantel test results. * *p* < 0.05; *** *p* < 0.001; ns: not significant. (**E**–**G**) GWAS results for KL (**E**), PH (**F**), and LUL (**G**). The blue line represents the suggestive significance threshold ($-{log}_{10}(P)\;=\;5$), corresponding to $P<1\times 10^{-5}$, while the red line indicates the genome-wide significant threshold ($-{log}_{10}(P)\approx 7.3$), corresponding to $P<5\times 10^{-8}$).

To evaluate the contribution of genetic variation to phenotypic differentiation, genotypic distance (Figure 3A) and phenotypic distance matrices (Figure 3B) were constructed for all inbred lines. Mantel analysis revealed only a weak positive correlation between genotypic and overall phenotypic distances (Figure 3C).

Further Mantel tests were conducted between the genotypic distance matrix and phenotypic distance matrices for each of the ten traits. The results showed that only KL, PH, and LUL exhibited significant or highly significant positive correlations with genetic distance (Figure 3D).

Based on these signals, GWAS was performed for KL, PH, and LUL. At the whole-population level, no SNPs for KL reached the genome-wide significance threshold (Figure 3E). For PH, two highly significant loci were detected on chromosomes 6 and 7 (Figure 3F). For LUL, one highly significant locus was identified on chromosome 9 (Figure 3G). Detailed information for these three highly significant SNPs is presented in Table S1. The population structure correction and QQ plots for these three traits are presented in Figure S2A–C.

### 3.5. Phenotypic PCA and K-Means Clustering of Inbred Lines

**Figure 4:**
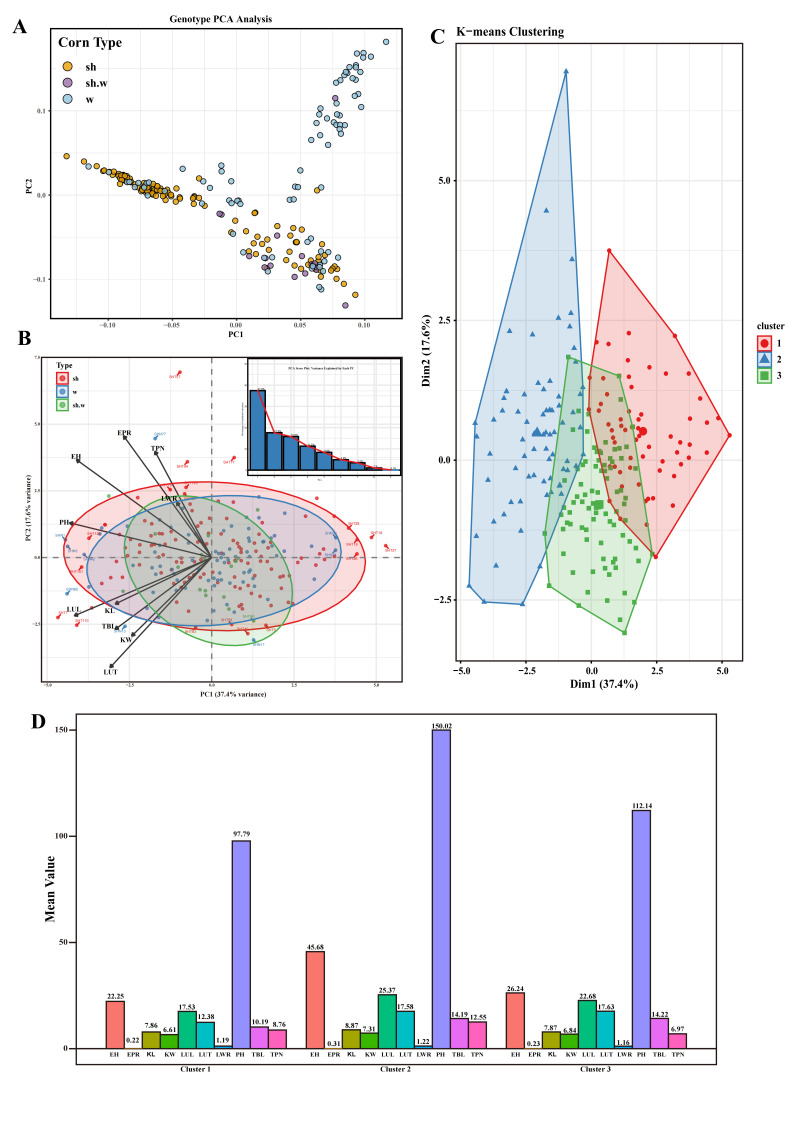
PCA and K-means clustering of 222 maize inbred lines. (**A**) PCA based on genotypic data. (**B**) PCA based on phenotypic data. (**C**) K-means clustering of phenotypic data into three clusters. (**D**) Mean trait values for each cluster.

Based on genotypic PCA, the 222 specialty maize inbred lines were grouped into three major clusters: the first cluster was predominantly composed of sweet corn, the second cluster was mainly waxy corn, and the third cluster contained a mixture of all three types (Figure 4A; detailed sample classification is provided in Table S1). In contrast, PCA based on ten phenotypic traits revealed substantial overlap among all inbred lines, indicating the absence of a clear population structure corresponding to the genotypic clusters (Figure 4B).

The biplot of phenotypic PCA loadings indicated that the first two principal components (PC1 and PC2) together explained 55.00% of the total phenotypic variation. Variation in PC1 was primarily driven by negative loadings of KL, KW, and tassel-related traits (LUT, LUL, TBL), whereas variation in PC2 was mainly associated with positive contributions from EPR, TPN, EH, LWR, and PH (detailed contribution rates of each trait are shown in Figure S3A).

To further dissect phenotypic variation, the optimal number of clusters was determined using both the silhouette method and the elbow method (Supplementary Figure S3B–C). K-means clustering of the phenotypic data divided the inbred lines into three clusters (Figure 4C; detailed classification is provided in Table S3). Cluster 1 exhibited a “short-stature, low-ear, compact tassel” phenotype, with PH (97.79 cm) and EH (22.25 cm) significantly lower than those of the other two clusters, and minimal values for TBL, LUL, and LUT, representing a typical compact dwarf type. Cluster 2 displayed a “tall-stature, large-ear, long-grain, well-developed tassel” phenotype, with the highest values across all measured traits, including PH of 150.02 cm, KL of 8.87 mm, and KW of 7.31 mm, indicative of vigorous vegetative growth and strong source capacity. Cluster 3 was characterized as “medium-stature, wide-grain, few tassel branches,” with intermediate PH (112.14 cm), the largest KW (6.84 mm), lowest LWR (1.16), and the lowest TPN (6.97), representing a typical wide-grain type with a distinct tassel branching pattern (Figure 4D).

### 3.6. Population Structure and Phylogenetic Analysis

**Figure 5:**
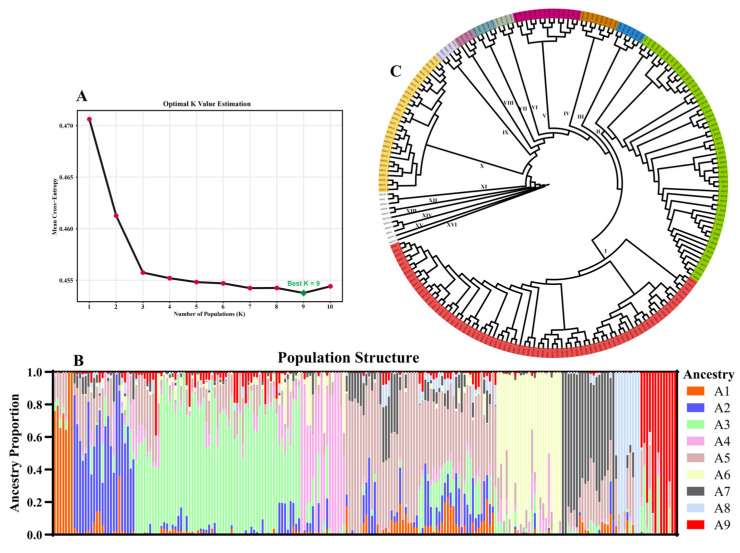
Population structure and phylogenetic analysis of 222 maize inbred lines. (**A**) Optimal K estimation. (**B**) Ancestry proportion of each line at K = 9. (**C**) Phylogenetic tree showing 16 major clades.

To further investigate the genetic composition and ancestral contributions within the population, we performed population structure and phylogenetic analyses. Based on population structure analysis, the optimal number of ancestral populations (K) was evaluated over a range of K = 1–10, with K = 9 providing the best fit (Figure 5A), indicating that the population comprises nine putative ancestral groups. The corresponding ancestry proportion plot revealed that most inbred lines contained contributions from multiple ancestral groups, reflecting a complex pattern of admixture (Figure 5B; detailed classification is provided in Table S4). Phylogenetic analysis further divided the 222 inbred lines into 16 major clades (Figure 5C). Among these, Clades I, II, V, and X constituted the backbone of the phylogenetic tree, each containing more than ten lines and collectively representing the majority of the population.

### 3.7. Core Germplasm Selection

**Figure 6:**
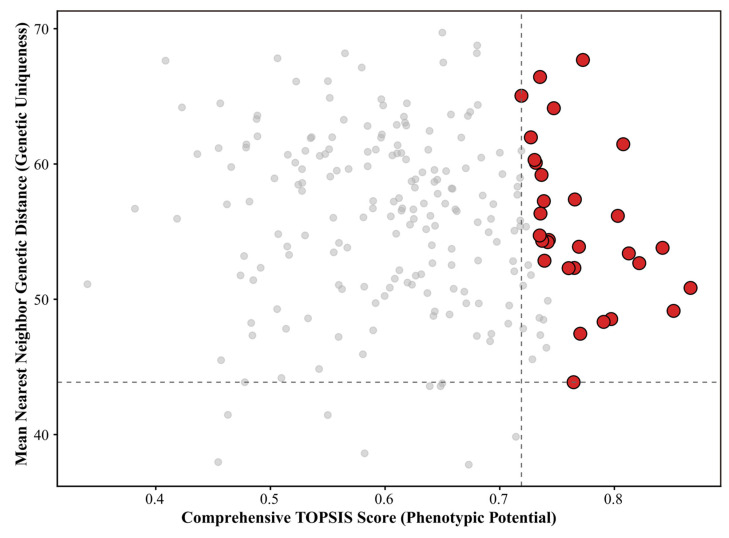
Scatter plot of MNND versus TOPSIS scores for 222 maize inbred lines. Each point represents an individual inbred line. The *x*-axis shows the comprehensive TOPSIS score and the *y*-axis shows the MNND. Red points indicate the top 30 lines selected as core germplasm based on the integrated MNND and TOPSIS scores. The dashed lines delineate the upper-right quadrant representing the top 50 lines based on TOPSIS scores (*D* values).

To comprehensively evaluate the genetic composition of the population and identify superior germplasm, the pairwise genotypic distance matrix for 222 inbred lines was constructed, from which the mean nearest-neighbor genetic distance (MNND) for each line was calculated. Based on ten traits (with kernel traits as positive indicators and plant architecture and tassel traits as negative indicators), CVs were used as weights for standardizing positive and negative indicators, and the phenotypic TOPSIS composite score (*D* value) was computed for each line. MNND and TOPSIS scores were integrated in a two-dimensional framework to generate the “MNND vs. TOPSIS score” scatter plot (Figure 6).

The top 50 lines based on *D* values were initially selected, and a weighted average (MNND 20%, *D* value 80%) was used to calculate a combined score, from which the top 30 lines were identified as the core germplasm (Table 2). The 30 selected lines exhibited a clear distribution pattern across texture types, with sweetcorn lines predominating (21 lines, 70.0%), waxy corn lines comprising 26.7% (8 lines), and sweet–waxy corn lines only 3.3% (1 line).

Considerable differentiation was observed in *D* values and MNND among the core lines. D values ranged from 0.7189 (SHN79, Rank 30) to 0.8664 (SHT18, Rank 1), with a range of 0.1475; the top five lines (SHT18, SHT27, SHT19, SHT45, SHT26) all exceeded a threshold of 0.81. MNND scores ranged from 43.87 (SHT38, Rank 15) to 67.69 (SHN69, Rank 10), with a range of 17.59, demonstrating substantial variation in genetic uniqueness.

Comparison of the core germplasm to the overall population mean revealed that the selected lines aligned closely with breeding objectives. For kernel traits, 60.0% (18 lines) of KL values exceeded the population mean (8.20 mm), with a maximum of 10.76 mm (SHT88); 50.0% (15 lines) and 53.3% (16 lines) of lines exceeded the mean for KW and LWR, respectively, reflecting effective capture of large-grain individuals. For plant architecture traits, core lines were predominantly compact and dwarf-type, with 66.7% (20 lines) exhibiting PH below the population mean (120.52 cm, minimum 58.00 cm, SHT18), 73.3% (22 lines) with EH below the mean (31.50 cm, minimum 6.17 cm, SHT5), and 63.3% (19 lines) with EPR below the mean. For tassel traits, although the overall range overlapped with the population mean, extreme over-proliferation was avoided. The top five lines consistently exhibited TPN values between 5.00 and 6.00, substantially lower than the population mean (9.31), while 60.0% (18 lines), 40.0% (12 lines), and 50.0% (15 lines) of lines had TBL, LUL, and LUT values at or below the population mean, without extreme elongation.

Population structure analysis indicated that the 30 core lines were distributed across nine clades, with Clade I as the primary genetic source (13 lines, 43.3%), followed by Clade II (6 lines, 20.0%), and the remaining seven clades collectively accounting for 36.7%. At the ancestral component level, Ancestry 5 dominated (12 lines, 40.0%), while Ancestry 4 and 7 each accounted for 13.3% (4 lines each); other components (Ancestry 2, 3, 8, 9, etc.) comprised 33.4% collectively. These distributions indicate that the core germplasm maintains key advantageous clades and ancestral contributions while incorporating broad genetic backgrounds, effectively avoiding genetic homogenization.

## 4. Discussion

Effectively minimizing environmental noise in multi-environment trials (MET) is crucial for accurately assessing genetic diversity. By integrating BLUP values across two environments, we successfully captured the intrinsic genetic variation among 222 specialty maize inbred lines. PH, EH, and TPN exhibited high coefficients of variation, indicating substantial intra-population genetic variability, which aligns with previous observations in diverse maize germplasm [17]. Notably, distinct phenotypic differentiation emerged among the sweet, waxy, and sweet–waxy subgroups: sweet maize displayed significantly greater KL and LWR, whereas waxy and sweet–waxy types exhibited higher EPR. This divergence likely reflects contrasting long-term breeding selection pressures—sweet maize improvement has historically emphasized kernel appearance and palatability, whereas waxy maize breeding has primarily targeted yield and processing adaptability, inadvertently preserving taller plant architectures in the latter [18].

SNP-based analysis revealed distinct genetic backgrounds among subgroups. Waxy maize showed the highest nucleotide diversity (π) and PIC, while sweet–waxy maize exhibited the highest average heterozygosity. Nine ancestral components and sixteen phylogenetic branches were identified, highlighting the multiple origins and complex pedigrees of these inbred lines, consistent with extensive germplasm exchange in modern maize breeding [19,20]. The weak overall correlation between phenotypic and genotypic distances, with only KL, PH, and LUL showing significant positive correlations, underscores the prevalence of convergent phenotypic selection and the polygenic nature of these agronomic traits. This suggests that BLUP-corrected phenotypes may not align linearly with genotypes [21].

GWAS analyses confirmed the complex genetic control of the examined traits. Highly significant SNPs associated with PH and LUL were detected on chromosomes 6, 7, and 9, aligning with previous GWAS reports that frequently identify major loci for plant architecture and tassel traits on these specific chromosomes, whereas stable KL-associated loci were absent. This likely reflects the polygenic, minor-effect nature of KL, where individual loci contribute minimally to phenotypic variance, as reported for other complex kernel morphology traits [22]. Capturing such variants typically requires larger populations or multi-omics integration. Conversely, the consistent detection of loci for plant architecture and tassel traits suggests control by major-effect QTLs, making them suitable targets for marker-assisted selection (MAS).

Combining phenotypic comprehensive evaluation (TOPSIS *D* value) with MNND provides a robust framework for core germplasm selection, mitigating environmental bias [23]. The 30 core inbred lines identified exhibit favorable trait combinations: superior kernel morphology and “dwarf, low-ear” plant architecture. In the context of high-density maize cultivation, this architecture enhances lodging resistance, light-use efficiency, and mechanized harvest adaptability [24], providing valuable parental material for hybrid breeding.

Although BLUP effectively reduces environmental noise, this study did not explicitly quantify genotype × environment (G × E) interactions. Future work should incorporate more diverse locations and multi-year trials to dissect G × E effects, distinguishing broadly versus specifically adapted germplasm. The limited number of sweet–waxy double-homozygous lines constrained deeper analyses of complex traits, emphasizing the need for expanded collection and characterization. For traits like KL, governed by minor-effect loci, future studies should integrate high-density markers, haplotype-based GWAS, and multi-trait joint prediction models. Coupling genomic analyses with transcriptomic or metabolomic data would further accelerate the genetic dissection of complex agronomic traits in specialty maize, advancing breeding from empirical to precision-design approaches.

## 5. Conclusions

This study comprehensively characterized the phenotypic and genetic diversity of 222 specialty maize inbred lines using both agronomic traits and high-density SNP markers. Significant variation was observed across kernel, plant, and tassel traits, with clear differentiation among sweet, waxy, and sweet–waxy subgroups. Population structure and phylogenetic analyses revealed complex ancestral contributions, with multiple clades and admixture patterns contributing to the genetic composition of the population. Integrating phenotypic potential (TOPSIS score) and genetic uniqueness (MNND), a set of 30 core inbred lines was identified that captures both desirable agronomic performance and broad genetic diversity. These core lines predominantly represent the target sweetcorn while maintaining representation of other texture types, and exhibit trait distributions aligned with modern breeding objectives, such as increased kernel size, short plant architecture, and moderate tassel development. The selected core germplasm provides a valuable resource for future breeding programs, facilitating targeted crosses, genomic studies, and germplasm conservation. Overall, this work demonstrates the effectiveness of combining phenotypic evaluation and genetic diversity metrics for rational core germplasm selection in specialty maize.

## Figures and Tables

**Table 1:** Descriptive statistics of phenotypic traits in 222 specialty maize inbred lines.

Trait	N	Mean	SD	Min	Max
KL/mm	222	8.2	1	4.14	10.76
KW/mm	222	6.93	0.81	3.33	8.67
LWR	222	1.19	0.08	1.05	1.47
PH/cm	222	120.52	29.07	58	191
EH/cm	222	31.5	13.93	6.17	78
EPR	222	0.25	0.08	0.05	0.82
TPN	222	9.31	4.83	1	29.67
TBL/cm	222	13.07	3.07	4.67	23.67
LUL/cm	222	22.1	4.47	9.77	37.67
LUT/cm	222	16.12	3.64	6.67	26.67

**Table 2:** Phenotypic, genetic, and phylogenetic characteristics of the top 30 core maize inbred lines.

ID	*D*_Value	MNND_Score	Rank	KL	KW	LWR	PH	EH	EPR	TPN	TBL	LUL	LUT	Clade	Ancestry
SHT18	0.8664	50.84	1	7.67	6.30	1.22	58.00	9.17	0.16	5.33	7.67	10.00	7.00	Clade II	Ancestry 2
SHT27	0.8517	49.14	2	6.86	6.24	1.10	66.13	8.17	0.12	5.67	7.53	9.77	7.34	Clade II	Ancestry 7
SHT19	0.8420	53.80	3	7.53	6.08	1.24	63.00	10.67	0.17	5.00	8.67	11.33	8.63	Clade II	Ancestry 2
SHT45	0.8217	52.67	4	8.28	6.42	1.29	61.67	11.67	0.19	6.00	9.00	14.67	10.67	Clade I	Ancestry 5
SHT26	0.8126	53.39	5	6.84	6.03	1.13	58.00	10.00	0.17	5.00	12.17	12.17	8.97	Clade II	Ancestry 7
SHN38	0.8077	61.46	6	7.33	6.13	1.19	92.00	14.00	0.16	3.67	7.17	13.50	11.67	Clade XI	Ancestry 2
SHN39	0.8030	56.17	7	7.49	6.22	1.20	73.00	15.00	0.20	5.67	7.67	13.17	10.50	Clade XII	Ancestry 4
SHT34	0.7972	48.54	8	7.67	6.52	1.18	71.67	15.00	0.21	3.00	9.67	15.00	13.33	Clade I	Ancestry 5
SHT24	0.7906	48.33	9	6.88	6.23	1.10	79.67	15.33	0.19	3.33	7.40	15.50	12.50	Clade II	Ancestry 7
SHN69	0.7726	67.69	10	7.26	6.69	1.09	70.17	13.00	0.18	4.67	11.83	16.33	13.33	Clade VIII	Ancestry 4
SHT28	0.7702	47.45	11	6.94	6.21	1.12	76.00	13.33	0.18	11.00	7.57	12.67	8.83	Clade I	Ancestry 7
SHT6	0.7691	53.88	12	8.10	7.30	1.11	87.27	6.67	0.08	8.33	11.17	20.27	13.87	Clade I	Ancestry 9
SHTN6	0.7655	57.38	13	8.91	7.46	1.20	76.67	10.67	0.14	4.33	11.97	22.80	18.83	Clade I	Ancestry 9
SHT44	0.7651	52.32	14	8.39	6.57	1.28	74.33	9.00	0.12	7.67	11.67	21.00	15.50	Clade I	Ancestry 5
SHT38	0.7644	43.87	15	7.63	6.34	1.21	86.67	17.00	0.20	2.00	11.27	17.93	15.40	Clade I	Ancestry 5
SHT17	0.7601	52.30	16	6.77	5.78	1.18	77.33	12.33	0.16	6.33	10.67	17.00	13.17	Clade II	Ancestry 5
SHN59	0.7472	64.12	17	8.05	7.10	1.13	100.00	23.00	0.23	5.67	8.00	16.33	12.33	Clade II	Ancestry 3
SHT20	0.7428	54.38	18	7.50	6.10	1.23	103.00	15.67	0.15	3.67	12.50	18.50	15.17	Clade I	Ancestry 5
SHT46	0.7418	54.23	19	8.77	6.62	1.33	101.67	13.67	0.13	3.33	13.00	22.23	19.33	Clade I	Ancestry 5
SHT37	0.7390	52.85	20	7.73	6.19	1.25	94.33	19.33	0.21	11.00	8.97	15.37	8.27	Clade V	Ancestry 5
SHN16	0.7385	57.25	21	7.70	7.28	1.06	79.33	19.00	0.24	1.67	12.50	19.67	17.33	Clade I	Ancestry 4
SHT58	0.7370	54.32	22	9.43	7.94	1.18	78.90	12.33	0.15	6.67	15.20	22.03	18.00	Clade I	Ancestry 2
SHN17	0.7365	59.19	23	7.97	7.43	1.07	85.67	11.93	0.14	2.00	14.50	22.17	20.97	Clade I	Ancestry 4
SHT5	0.7355	56.34	24	7.54	6.30	1.19	117.33	6.17	0.05	3.00	14.83	22.37	19.07	Clade II	Ancestry 5
SHT108	0.7351	66.43	25	8.69	7.19	1.21	111.00	16.67	0.15	3.00	12.00	22.33	18.00	Clade III	Ancestry 3
SHT47	0.7349	54.72	26	8.28	6.44	1.28	100.67	16.00	0.16	3.33	11.00	22.47	19.17	Clade VI	Ancestry 8
SHT88	0.7314	60.09	27	10.76	8.31	1.30	107.67	21.33	0.20	4.33	14.00	22.67	17.33	Clade II	Ancestry 5
SHT60	0.7304	60.30	28	7.05	6.14	1.15	94.67	11.67	0.12	3.33	19.10	16.17	15.33	Clade X	Ancestry 5
SHN60	0.7272	61.97	29	7.55	6.62	1.14	90.33	33.67	0.37	3.33	7.83	14.87	10.67	Clade X	Ancestry 3
SHN79	0.7189	65.04	30	8.57	8.10	1.06	131.67	20.00	0.15	3.33	11.67	20.33	16.83	Clade I	Ancestry 5
Mean				8.20	6.93	1.19	120.52	31.50	0.25	9.31	13.07	22.10	16.12		

## Data Availability

The original contributions presented in this study are included in the article/Supplementary Materials. Further inquiries can be directed to the corresponding author.

## Supplementary Materials

The following supporting information can be downloaded at: https://www.mdpi.com/article/10.3390/genes17050568/s1, Figure S1: Proportion of phenotypic variance explained by different components for each trait; Figure S2: QQ plots of GWAS; Figure S3: PCA variable contributions and determination of optimal K for K-means clustering; Table S1: Summary statistics of the extremely significant SNPs associated with Plant Height (PH) and LUL; Table S2: Genotype_PCA_Results; Table S3: kmeans_cluster_result; Table S4: Ancestry proportions of 222 maize inbred lines at K = 9.

## References

[1] Li X., Li Y., Sun Y., Li S., Cai Q., Li S., Sun M., Yu T., Meng X., Zhang J. (2025). Integrating Genetic Diversity and Agronomic Innovations for Climate-Resilient Maize Systems. Plants.

[2] Sidahmed H., Vad A., Nagy J. (2025). Advances in Sweet Corn (*Zea mays* L. Saccharata) Research from 2010 to 2025: Genetics, Agronomy, and Sustainable Production. Agronomy.

[3] Liu H.-J., Liu J., Zhai Z., Dai M., Tian F., Wu Y., Tang J., Lu Y., Wang H., Jackson D. (2025). Maize2035: A Decadal Vision for Intelligent Maize Breeding. Mol. Plant.

[4] Yang Q., Guo Z., Xu Y., Wang Y. (2024). Genetic Diversity and Population Structure of Sweet Corn in China as Revealed by mSNP. Mol. Breed..

[5] Dang D., Guan Y., Zheng H., Zhang X., Zhang A., Wang H., Ruan Y., Qin L. (2023). Genome-Wide Association Study and Genomic Prediction on Plant Architecture Traits in Sweet Corn and Waxy Corn. Plants.

[6] Dube S.P., Sibiya J., Kutu F. (2023). Genetic Diversity and Population Structure of Maize Inbred Lines Using Phenotypic Traits and Single Nucleotide Polymorphism (SNP) Markers. Sci. Rep..

[7] Danecek P., Auton A., Abecasis G., Albers C.A., Banks E., DePristo M.A., Handsaker R.E., Lunter G., Marth G.T., Sherry S.T. (2011). 1000 Genomes Project Analysis Group. The Variant Call Format and VCFtools. Bioinformatics.

[8] Chang C.C., Chow C.C., Tellier L.C., Vattikuti S., Purcell S.M., Lee J.J. (2015). Second-Generation PLINK: Rising to the Challenge of Larger and Richer Datasets. Gigascience.

[9] Yang R., Wu X., Bai Y., He Y., Chang S., Hai L. (2025). Genome-Wide SNP Analysis Reveals Population Structure and Genetic Diversity in Lycium Ruthenicum Murr. Plants.

[10] Azam M.G., Sarker U., Hossain M.A., Mahabubul Alam A.K.M., Islam M.R., Hossain N., Alamri S. (2024). Phenotypic Diversity in Qualitative and Quantitative Traits for Selection of High Yield Potential Field Pea Genotypes. Sci. Rep..

[11] Quilodrán C.S., Currat M., Montoya-Burgos J.I. (2025). Benchmarking the Mantel Test and Derived Methods for Testing Association between Distance Matrices. Mol. Ecol. Resour..

[12] Zhou X., Stephens M. (2012). Genome-Wide Efficient Mixed-Model Analysis for Association Studies. Nat. Genet..

[13] Pritchard J.K., Stephens M., Donnelly P. (2000). Inference of Population Structure Using Multilocus Genotype Data. Genetics.

[14] Chiemeke F.K., Olasanmi B., Agre P.A., Mushoriwa H., Chigeza G., Abebe A.T. (2024). Genetic Diversity and Population Structure Analysis of Soybean [Glycine Max (L.) Merrill] Genotypes Using Agro-Morphological Traits and SNP Markers. Genes.

[15] Dos Santos C.C., de Andrade L.R.B., do Carmo C.D., de Oliveira E.J. (2023). Development of Cassava Core Collections Based on Morphological and Agronomic Traits and SNPS Markers. Front. Plant Sci..

[16] Duc N.T., Tuan P.Q., Nguyet Anh N.T., Van Liet V. (2024). Phenotypic Diversity and Selection of Superior Tropical Sweetcorn Inbred Lines by Multivariate Method and Combining Ability Analysis. Ecol. Genet. Genom..

[17] Zeng T., Meng Z., Yue R., Lu S., Li W., Li W., Meng H., Sun Q. (2022). Genome Wide Association Analysis for Yield Related Traits in Maize. BMC Plant Biol..

[18] Qu J., Yu D., Gu W., Khalid M.H.B., Kuang H., Dang D., Wang H., Prasanna B., Zhang X., Zhang A. (2024). Genetic Architecture of Kernel-Related Traits in Sweet and Waxy Maize Revealed by Genome-Wide Association Analysis. Front. Genet..

[19] Yu J., Holland J.B., McMullen M.D., Buckler E.S. (2008). Genetic Design and Statistical Power of Nested Association Mapping in Maize. Genetics.

[20] Gore M.A., Chia J.-M., Elshire R.J., Sun Q., Ersoz E.S., Hurwitz B.L., Peiffer J.A., McMullen M.D., Grills G.S., Ross-Ibarra J. (2009). A First-Generation Haplotype Map of Maize. Science.

[21] Zhou X., Stephens M. (2014). Efficient Multivariate Linear Mixed Model Algorithms for Genome-Wide Association Studies. Nat. Methods.

[22] Ma J., Wang L., Cao Y., Wang H., Li H. (2021). Association Mapping and Transcriptome Analysis Reveal the Genetic Architecture of Maize Kernel Size. Front. Plant Sci..

[23] Pessoa-Filho M., Rangel P.H., Ferreira M.E. (2010). Extracting Samples of High Diversity from Thematic Collections of Large Gene Banks Using a Genetic-Distance Based Approach. BMC Plant Biol..

[24] Wang C., He W., Li K., Yu Y., Zhang X., Yang S., Wang Y., Yu L., Huang W., Yu H. (2025). Genetic Diversity Analysis and GWAS of Plant Height and Ear Height in Maize Inbred Lines from South-East China. Plants.

