# Supplementary material for: Integrating Multi-Environment Phenotypes and Genome-Wide Variation to Evaluate Diversity and Identify Representative Germplasm in Specialty Maize
Source: Genes (Basel). 2026 May 17;17(5):568. doi: 10.3390/genes17050568 (PMC13205148; doi:10.3390/genes17050568)
Supplement: Supplementary file 1 [file genes-17-00568-s001.zip › FigureS1 S2 S3.pdf]

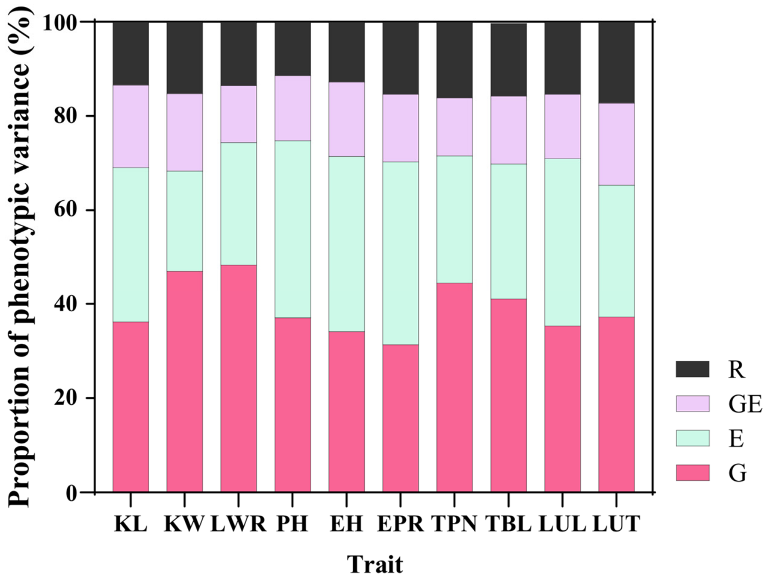

Figure S1. Proportion of phenotypic variance explained by different components for each trait. R: Residual; GE: Genotype × Environment interaction; E: Environment; G: Genotype.

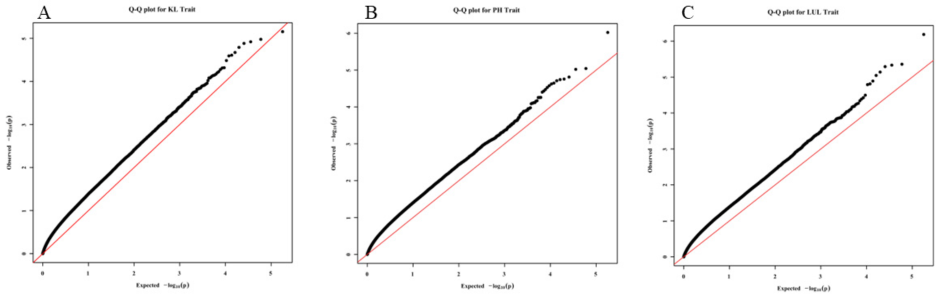

Figure S2. QQ plots of GWAS for KL (A), PH (B), and LUL (C).

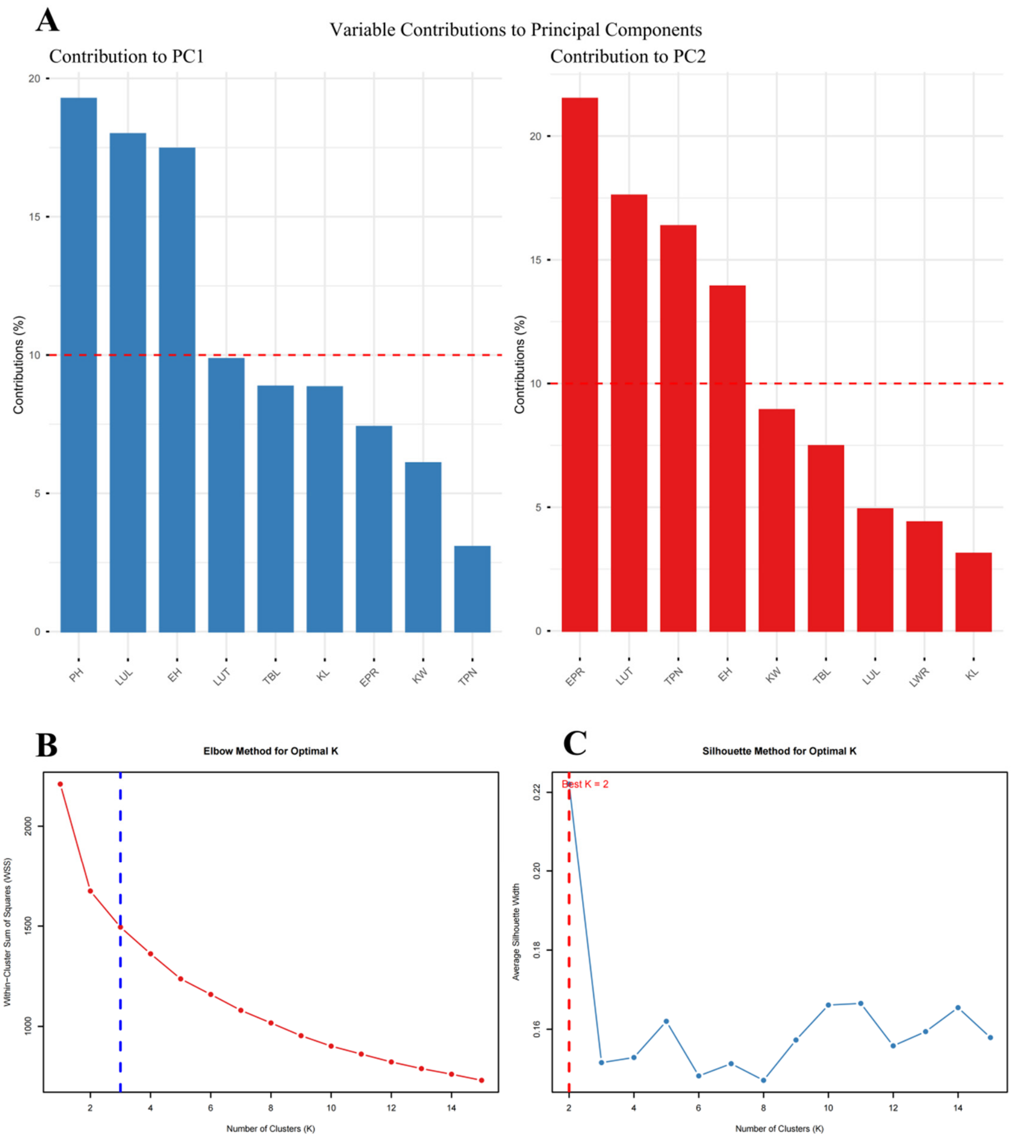

Figure S3. PCA variable contributions and determination of optimal K for K-means clustering.

(A) Contributions of traits to PC1 and PC2.

(B) Elbow method for optimal K.

(C) Silhouette method for optimal K.
