# Supplementary material for: Integrating Multi-Environment Phenotypes and Genome-Wide Variation to Evaluate Diversity and Identify Representative Germplasm in Specialty Maize
Source: Genes (Basel). 2026 May 17;17(5):568. doi: 10.3390/genes17050568 (PMC13205148; doi:10.3390/genes17050568)
Supplement: Supplementary file 1 [file genes-17-00568-s001.zip › Table S1-Summary statistics of the extremely significant SNPs associated with Plant Height (PH) and LUL.pdf]

Table S1. Summary statistics of the extremely significant SNPs associated with PH and LUL

Trait	chr	rs	ps	n_miss	allele1	allele0	af	beta	se	logl_H1	l_remle	l_mle	p_wald	p_lrt	p_score
PH	6	.	53739356	0	C	G	0.117	-25.84036	4.438873	-1039.239	0.1853859	0.1790083	2.05E-08	2.60E-07	4.85E-06
	7	.	167306513	11	A	C	0.339	-11.80552	2.087019	-1039.505	0.2241457	0.2210797	4.77E-08	3.43E-07	5.17E-06
LUL	9	.	50122322	10	T	C	0.068	-4.132546	0.7288366	-631.897	0.000425462	1.00E-05	4.46E-08	1.94E-06	1.72E-05

Note：Trait, the target trait analyzed (PH or LUL); chr, chromosome number; rs, SNP identifier; ps, physical position (bp); n_miss, number of missing genotypes for the SNP; allele1, minor allele; allele0, major allele; af, minor allele frequency (MAF); beta, estimated additive effect of the minor allele on the phenotype; se, standard error of the beta estimate; logl_H1, maximized log-likelihood under the alternative hypothesis; l_remle, log-restricted maximum likelihood under the null model; l_mle, maximized log-likelihood under the null model; p_wald, p-value from the Wald test; p_lrt, p-value from the likelihood ratio test; p_score, p-value from the score test.
